# Supplementary figures and images for: Characteristics and evolution of hemagglutinin and neuraminidase genes of Influenza A(H3N2) viruses in Thailand during 2015 to 2018
Source: PeerJ. 2024 Jun 3;12:e17523. doi: 10.7717/peerj.17523 (PMC11155671; doi:10.7717/peerj.17523)

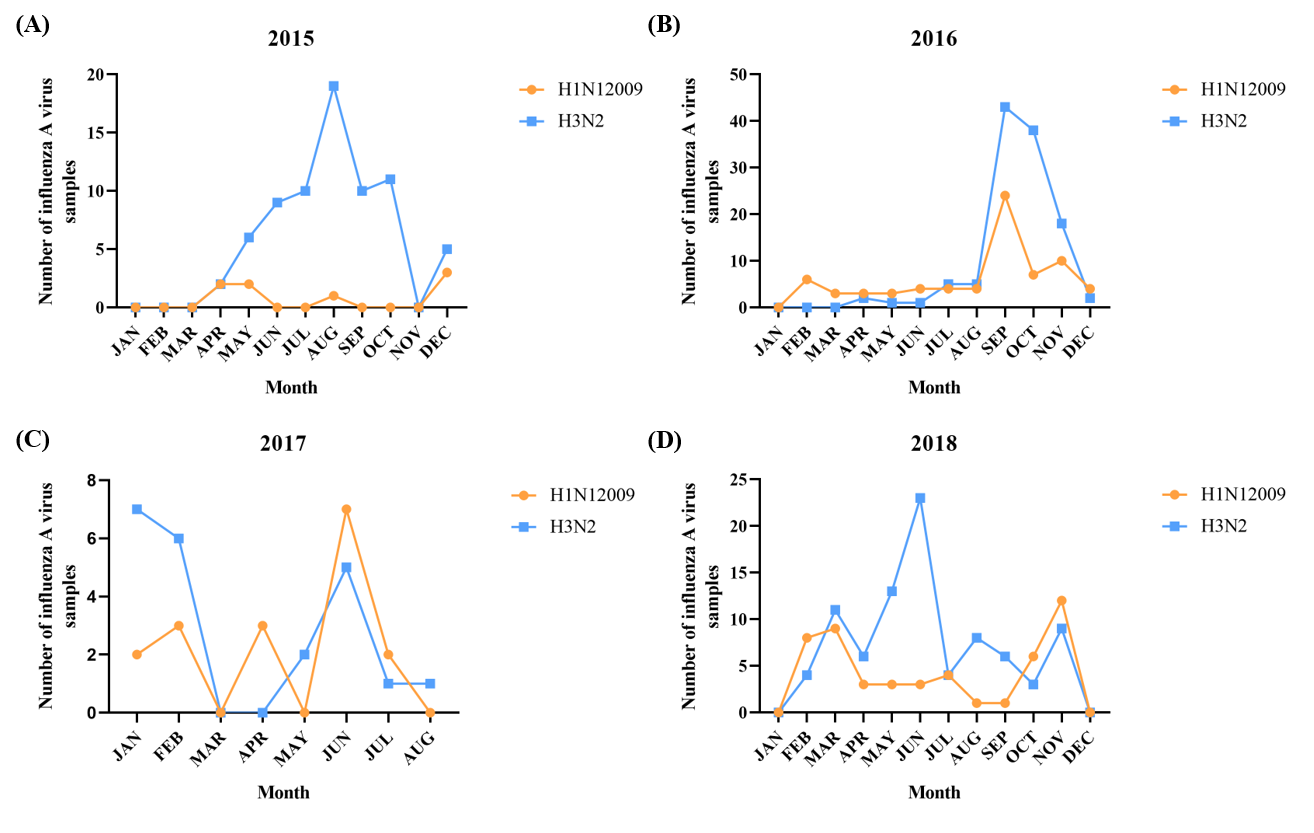

Supplement: Supplemental Information 1 — A total of 443 influenza A positive samples from 2015-2018 were subtyped using multiplex real-time RT-PCR, and the subtyped samples were reported monthly. (A) the influenza A-positive samples collected in 2015, (B) 2016, (C) 2017, and (D) 2018. Orange represent A(H1N1)pdm2009 and blue; A(H3N2). *In 2017, the result showed the specimen collection from January to August due to no sample being available from September to December. [file peerj-12-17523-s001.png]

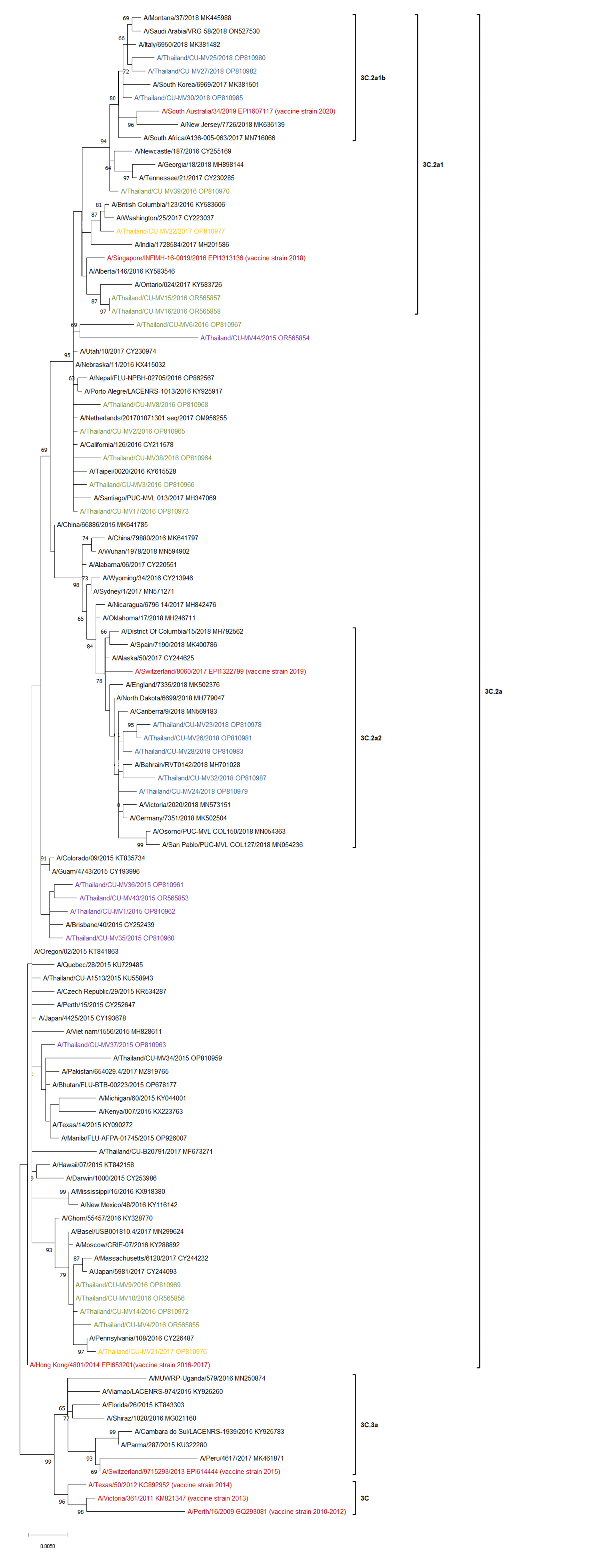

Supplement: Supplemental Information 2 — The phylogenetic tree of the hemagglutinin (HA) gene of the influenza A(H3N2) viruses from 2015 to 2018, vaccine strains, and circulating viruses from Africa, Asia, Europe, North America, South America, and Oceania continent was constructed using the Maximum Likelihood method based on the Hasegawa-Kishino-Yano and Gamma distributed (HKY+G) substitution model with 1,000 bootstrap replication. Red represents the WHO recommended influenza vaccine strains in the southern hemisphere from 2010 to 2018, purple represents the influenza A(H3N2) viruses from 2015, green; 2016, yellow; 2017, and blue; 2018. [file peerj-12-17523-s002.png]
